# Alternative-Splicing in the Exon-10 Region of GABA_A_ Receptor β_2_ Subunit Gene: Relationships between Novel Isoforms and Psychotic Disorders

**DOI:** 10.1371/journal.pone.0006977

**Published:** 2009-09-18

**Authors:** Cunyou Zhao, Zhiwen Xu, Feng Wang, Jianhuan Chen, Siu-Kin Ng, Pak-Wing Wong, Zhiliang Yu, Frank W. Pun, Lihuan Ren, Wing-Sze Lo, Shui-Ying Tsang, Hong Xue

**Affiliations:** Department of Biochemistry and Applied Genomics Center, Fok Ying Tung Graduate School, The Hong Kong University of Science & Technology, Clear Water Bay, Hong Kong, China; University of Muenster, Germany

## Abstract

**Background:**

Non-coding single nucleotide polymorphisms (SNPs) in *GABRB2*, the gene for β_2_-subunit of gamma-aminobutyric acid type A (GABA_A_) receptor, have been associated with schizophrenia (SCZ) and quantitatively correlated to mRNA expression and alternative splicing.

**Methods and Findings:**

Expression of the Exon 10 region of *GABRB2* from minigene constructs revealed this region to be an “alternative splicing hotspot” that readily gave rise to differently spliced isoforms depending on intron sequences. This led to a search in human brain cDNA libraries, and the discovery of two novel isoforms, β_2S1_ and β_2S2_, bearing variations in the neighborhood of Exon-10. Quantitative real-time PCR analysis of postmortem brain samples showed increased β_2S1_ expression and decreased β_2S2_ expression in both SCZ and bipolar disorder (BPD) compared to controls. Disease-control differences were significantly correlated with SNP rs187269 in BPD males for both β_2S1_ and β_2S2_ expressions, and significantly correlated with SNPs rs2546620 and rs187269 in SCZ males for β_2S2_ expression. Moreover, site-directed mutagenesis indicated that Thr^365^, a potential phosphorylation site in Exon-10, played a key role in determining the time profile of the ATP-dependent electrophysiological current run-down.

**Conclusion:**

This study therefore provided experimental evidence for the importance of non-coding sequences in the Exon-10 region in *GABRB2* with respect to β_2_-subunit splicing diversity and the etiologies of SCZ and BPD.

## Introduction

**Figure 1:**
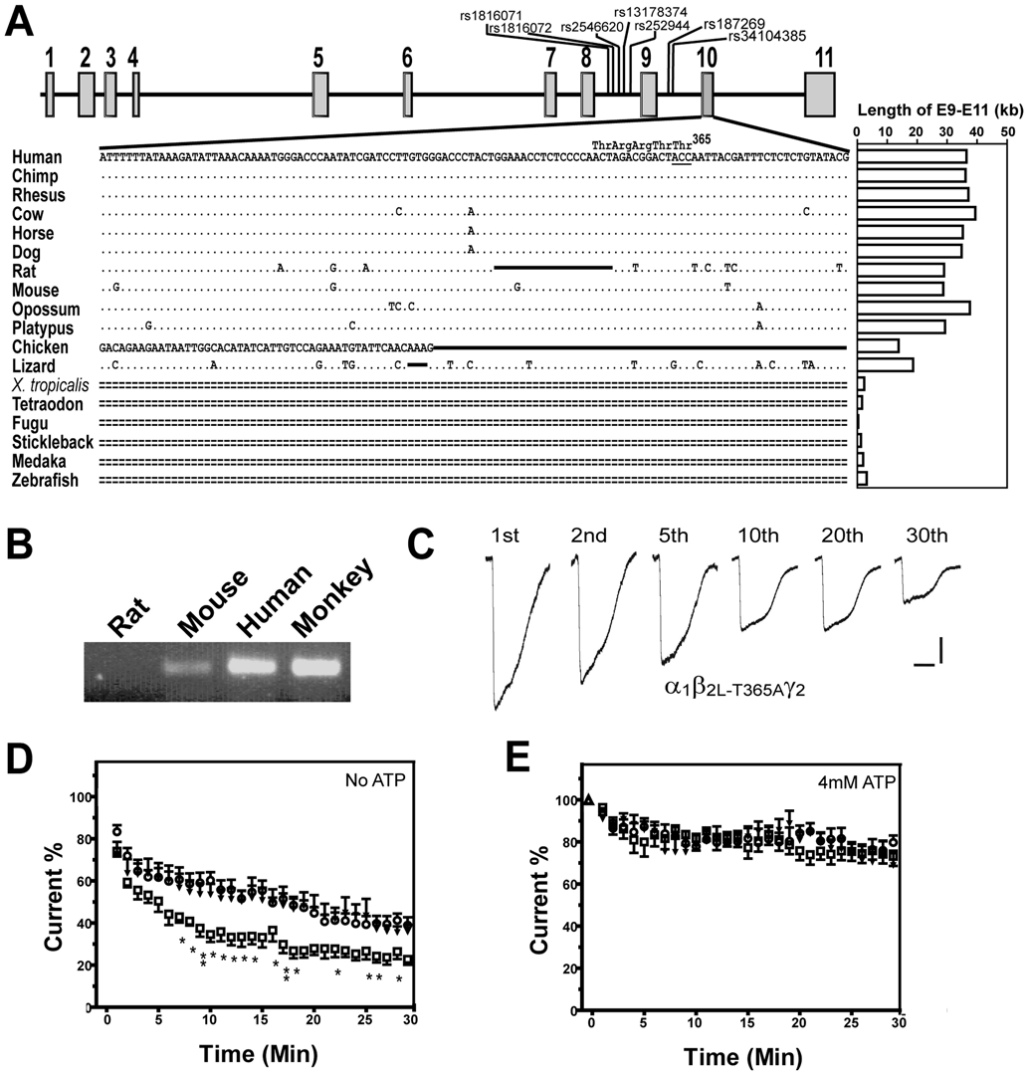
Characterizations of Exon 10 of *GABRB2*. (A) The exonic structure of human *GABRB2* showing positions of the tagging SNPs, and the alignment of its Exon 10 with corresponding sequences in other vertebrates (from UCSC Genome Browser). The lengths of the sequences corresponding to the intervening sequences between E9 and E11 in the different species are indicated on the right. Dots represent identities; single line, deletions; double-dashed line,unalignable bases. Exons are shown as numbered boxes. (B) RT-PCR analyses of Exon 10 expressions from rat, mouse, human and monkey brain cDNA libraries with the common forward primer F2 against Exon 9 and reverse primer R4.2 against Exon 10 (Table S4). (C) GABA-potentiated current rundowns of GABA_A_ receptors containing α_1_, γ_2_ and β_2L-T365A_. (D) The measured currents upon successive additions of GABA expressed as % of the peak current elicited by the first addition of GABA. Significantly different responses between the α_1_β_2L-T365A_γ_2L_ (▾) and α_1_β_2L_γ_2L_ (□) receptors are marked by single (*P*<0.05) or double (*P*<0.01) asterisks. (E) Same as part D with 4 mM ATP infusion.

γ-Aminobutyric acid (GABA) is the major inhibitory amino acid neurotransmitter in the vertebrate nervous system. The fast synaptic inhibition is mediated by the opening of a chloride channel formed by the GABA_A_ receptors. The GABA_A_ receptors are also clinically relevant drug targets for anti-convulsant, anxiolytic and sedative-hypnotic agents. Subtypes of GABA_A_ receptors are assembled from pentameric combinations of α_1_-α_6_, β_1_-β_3_, γ_1_-γ_3_, ρ_1_-ρ_3_, ε, δ and π subunits [1], [2]. The molecular heterogeneity of GABA_A_ receptor subunits is further increased by the alternative splicing of some subunit mRNAs, producing at least two isoforms of each of the α_6_, β_2_, β_3_ and γ_2_ subunits [3]. Most GABA_A_ receptors are composed of two α subunits, two β_2_ subunits and one γ subunit [4]. The β_2_ subunit gene products of human *GABRB2* expressed from cDNA library are found in two alternatively spliced isoforms, the short form β_2S_ and the long form β_2L_. The inclusion of an extra 38-amino acid Exon 10 in β_2L_ but not in β_2S_ in an intracellular loop brings with it a potential phosphorylation site at Thr^365^ for calmodulin-dependent protein kinase II (Figure 1A)[5].

Schizophrenia (SCZ) and bipolar disorder (BPD) are major psychotic disorders with a significant genetic component in their complex etiology [6]. *GABRB2*, which encodes the β_2_ subunit of GABA_A_ receptors, contains single nucleotide polymorphisms (SNPs) in non-coding sequences in the Intron 8-Intron 9 region that have been associated with SCZ [7]–[10]. Moreover, the expressions of β_2S_ and β_2L_ mRNA were found to be differentially decreased in the dorsolateral prefrontal cortex (DLPFC) of SCZ brains by real-time quantitative PCR, and these altered expressions were correlated with the SCZ-associated SNPs rs1816071 and rs1816072 in SCZ subjects carrying the heterozygous genotypes [11]. Since GABA_A_ receptors containing β_2L_ underwent a steeper current run-down than those containing β_2S_ under conditions of ATP depletion induced by repeated GABA-stimulation [11], these findings pointed to relationships between the alternate splicing of the Exon 10 region with both GABA current regulation and schizophrenia. It is therefore the objective of the present study to examine the factors determining the alternative splicing of Exon 10, and how the different β_2_ isoforms might be correlated with the major psychotic disorders.

## Results

### Variability of Exon 10 splicing in vertebrates

Based on sequence alignments, Exon 10 is readily identified in mammals and lizard, but not in chicken, frog (*X. tropicalis*) or fishes (Figure 1A). In contrast, Exon 9 and Exon 11 are present in all of these vertebrate species (Figure S1). Notably, expressions of Exon 10 have been reported for both humans [5] and chicken [12], [13], even though the Exon 10 sequence expressed in chicken bears little resemblance to that in mammals. In the present study, expression of Exon 10 was detected in rhesus monkey and mouse brains but not in rat brain (Figure 1B). The full-length β_2L_ coding sequences amplified from monkey and mouse brain cDNA libraries also confirmed the presence of β_2L_ in these two species (Figure S2). Thus there exist considerable variations among the vertebrates with respect to Exon 10 expression.

The species variations in Exon 10 expression are paralleled by variations in the length of the intervening gene segment between Exon 9 and Exon 11(Figure 1A, right), which increases from <3,000 bp in the fishes to 14,000–19,000 bp in non-mammalian land vertebrates, and >28,000 bp in marsupial and placental mammals. Furthermore, this region of the gene is readily blocked by RepeatMasker, which identifies DNA sequences containing interspersed repeats and low sequence complexity [14]. This suggests that insertions and expansions of repeat elements could be a significant contributing factor to the sharp increase in Exon 9-Exon 11 length from the lower vertebrates to the higher vertebrates. In fact, the sequence from 85-bp upstream to 66-bp downstream of Exon 10 has been annotated as a candidate LINE3, viz. a member of the Long Interspersed Nucleotide Element family (UCSC GenomeBrowser: http://genome.ucsc.edu/cgi-bin/hgTracks). Evolutionary changes in the lengths of the gene segments corresponding to the other human introns in *GABRB2* are shown in Figure S3. Since only gene but not cDNA sequences are available in various non-human species, their exact exonic structures yet remain to be ascertained.

### Expressions of human Exon 10 region from minigenes

**Figure 2:**
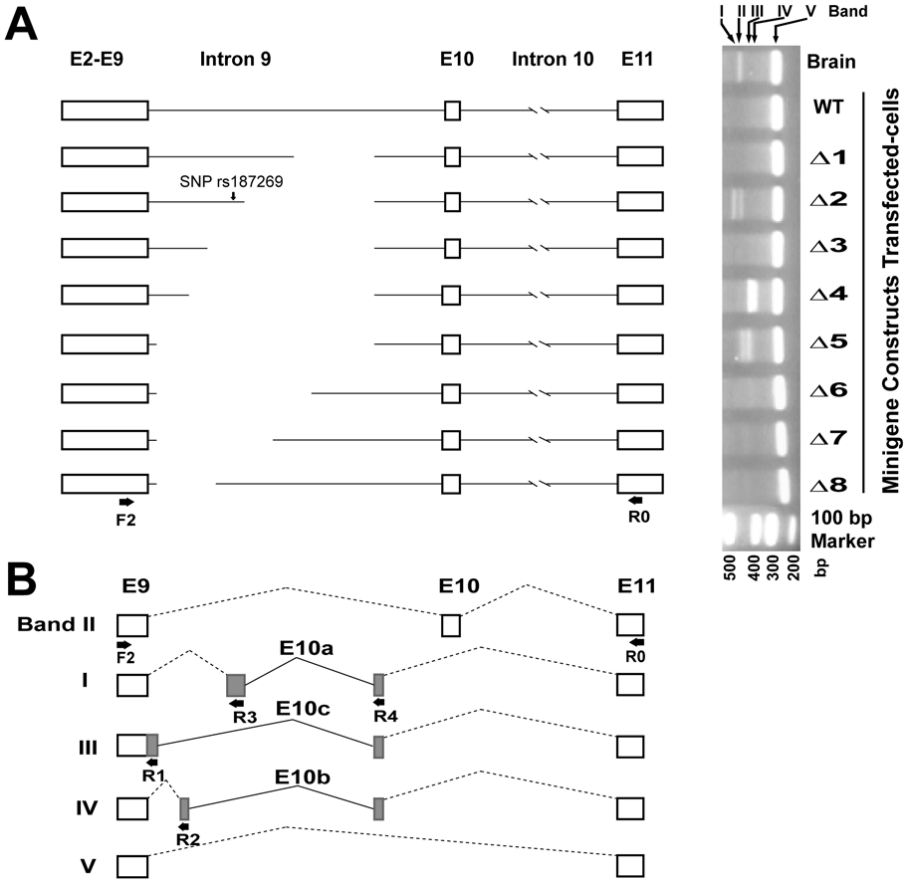
Effects of intronic sequences on Exon 10 expression in minigene constructs. (A) Minigenes of human *GABRB2* and their expressions in transfected cells. Left panel shows the structures of the minigenes; right panel shows the RT-PCR products amplified, using primers F2 and R0, from RNA derived from human brain or from minigene transfected-cells. Minigene WT consisted of E2-9, I9, E10, I10 (with a deletion ranging from 1208 bp downstream of E10 to 1126 bp upstream of E11, as indicated by the gap), and E11. Minigenes Δ1–Δ8 each carried a further deletion (shown as a gap) within I9. The determined sequences of Bands I–V are shown in Figure S4. (B) The proposed splicing origins of Bands I–V. Band II contained [3′portion of E9]-[E10]-[5′portion of E11]; Band V contained [3′portion of E9]-[5′portion of E11]. Band I contained the 3′-portion of E9, a replacement of E10 by E10a, which was derived from a joining of the two grey boxes, plus the 5′-portion of E11; Band III contained the 3′-portion of E9, a replacement of E10 by E10b derived from a joining of the two grey boxes, plus the 5′-portion of E11; Band IV was similar to Band I, except that one of the two grey boxes was different. The dotted bent lines between adjacent exons indicate fusion brought about by splicing, and the solid bent lines indicate fusion brought about by deletion in the minigene construct.

In order to identify some of the effects of intronic sequences flanking Exon 10 on the splicing of the region between Exon 9 and Exon 11, minigene constructs containing Exons 2–9, a varying length of Intron 9, Exon 10, an shortened Intron 10, and Exon 11 were constructed and expressed in Human embryonic kidney (HEK) 293 cells (Figure 2). This wild-type (WT) minigene construct containing a full length Intron 9, was unfavorable toward the generation of Exon 10, as indicated by the absence of a significant Exon 10-containing Band II DNA in the WT gel slot (Lane 2, Figure 2A). Similarly, the Δ1, Δ3, Δ6, Δ7 and Δ8 minigenes, each containing a shortened Intron 9, also did not give rise to any Band II DNA. Surprisingly, when the sequence between bp 1,451 and 3,513 in Intron 9 was further deleted from Δ1 to yield Δ2, a significant Exon 10-containing Band II was obtained, along with a Band I which contained a novel Exon 10a consisting of two segments of base sequences located between the E9 and E10 sequences (Figure 2B and Figure S4). When Δ2 underwent a further shortening of Intron 9 in the direction toward Exon 9 to yield Δ3, removing 451 bases including the rs187269 SNP site, both Exon 10 and Exon 10a disappeared. With still further shortening of Δ3 to yield Δ4, a different novel Exon 10b appeared in the form of Band IV. Shortening of Δ4 to yield Δ5 led to a re-emergence of the Exon 10-containing Band II along with a novel Exon 10c in the form of Band III. These observed exonal variations encoded by minigenes WT and Δ1–Δ8 pointed to the complexity of Exon 10 expression, in keeping with the relative ease of generation of new splicing forms in this region.

### Identification of potential isoforms from human brain cDNA

**Figure 3:**
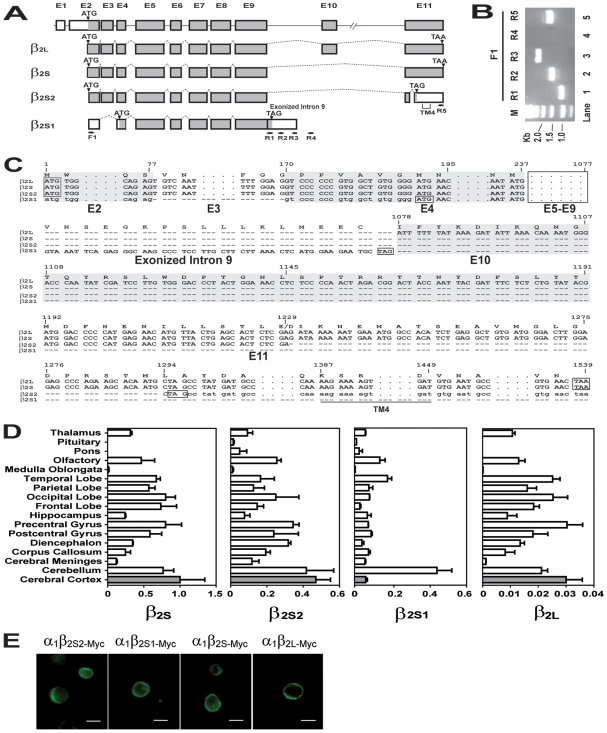
Novel splicing isoforms of the human GABA_A_ receptor β_2_ subunit. (A) Exonal structures of four different β_2_ subunit isoforms in human brain. Line 1 shows *GABRB2* structure with exons indicated by boxes (shaded for coding regions, and unshaded for non-coding regions). Lines 2–5 show the exonal compositions of alternatively spliced forms of β_2_. (B) EB-stained PCR products amplified from human brain cDNA library. Lane M, DNA standards; Lanes 1–5, PCR products obtained using forward primer F1 with reverse primers R1- R5 (Table S4), respectively. (C) Alignment of the cDNA sequences of the four isoforms. Base-pair numbering was based on β_2L_ cDNA. Bases in uppercase represent coding sequences, and those in lowercase represent non-coding sequences. Exons E2-E12 are shown in alternating shaded/unshaded backgrounds. The dotted lines represent intervening sequences that are not shown in detail. The dashed lines represent gapped/absent sequences. The start-codon ATG and stop-codons TAG or TAA are boxed. The italicized *GAC* spanning bp 1228–1294 incorporated a frame-shift that generated a 3′ TAG termination codon for β_2S2_. The fourth transmembrane domain (TM4) is marked in part A and underlined in part C. (D) The expression levels of β_2S_, β_2S2_, β_2S1_ and β_2L_ in different human brain regions relative to β_2S_ level in cerebral cortex were determined by means of real-time PCR. The amounts of different isoforms in the cerebral cortex (grey columns) relative to β_2S_ were: β_2S2_, 0.47; β_2S1_, 0.06; and β_2L_ 0.03. (E) Surface location of recombinant GABA_A_ receptors containing different β_2_ subunits (scale bars = 10 µm).

The results described in Figure 2 revealed splicing variants that generated three novel exons from the minigenes Δ2, Δ4 and Δ5: Exon 10a from Δ2, Exon 10b from Δ4, and Exon 10c from Δ5. In view of this observed propensity of the Exon 9-Exon 11 region to give rise to these novel exons in minigene transfected cells, it becomes of interest to determine whether the novel exons of this kind might also be expressed in the human brain. Accordingly, a human brain cDNA library (Resgen, Invitrogen) was PCR-amplified with primers F1 (forward), and R1, R2, R3, R4 and R5 (reverse) designed to amplify such novel exons, as illustrated in Figure 2B and Figure 3A. The amplified PCR products shown in Figure 3B were cloned into the pMD-18T vector and sequenced. The sequencing results in Figure 3C showed that the Lane 1, Lane 2 and Lane 3 DNAs were all derived from a cDNA encoding a novel isoform β_2S1_ that spanned from the F1 primer to a point between the R3 and R4 primers. The absence of a visible DNA band in Lane 4 indicated the lack of significant F1-R4 PCR product. In Lane 5, a F1-R5 PCR reaction gave rise to three distinct DNA sequences corresponding to the known long (β_2L_) and short (β_2S_) isoforms together with a novel isoform β_2S2_.

The mRNA of the novel isoform β_2S1_, defined by the PCR primers F1 and R3, was at least 2,470 bp in length. It contained a 3′-extended Exon 9 from Intron 9 retention and was devoid of Exons 3, 10 and 11, resulting in a 314-amino acid polypeptide. In contrast, the mRNA of β_2S2_ was at least 1,362 bp in length, was devoid of Exon 10, and contained an Exon 11 shortened by a 64-bp deletion that brought about a frameshift-generated TAG to result in a 373-amino acid polypeptide (Figure 3C). Figure 3D shows the relative mRNA expression levels of β_2S1_ and β_2S2_ in different human brain regions. Their relative amounts in the cerebral cortex were: β_2S_>β_2S2_>>β_2S1_>β_2L_.

### Electrophysiological properties of recombinant receptors

The cell surface location of recombinant GABA_A_ receptors containing different isoforms of β_2_ together with α_1_ subunits upon expression in HEK293 cells was indicated by immunofluorescence staining (Figure 3E). The electrophysiological responses of these receptors to GABA stimulation are shown in Figure S5A. The half-maximal GABA effective concentration (EC_50_) of the α_1_β_2_-myc receptors containing different isoforms of β_2_ ranged from 2.5–3.4 µM (Table 1 and Figure S5B).

**Table 1:** Electrophysiological parameters of splicing isoforms.

Isoform	N	EC_50_(µM)	Hill coefficient	I_max_(nA)
α_1_β_2S_-myc	5	2.5±0.3	1.8±0.4	867±83
α_1_β_2S1_-myc	8	2.6±0.1	2.0±0.2	696±90
α_1_β_2L_-myc	6	3.1±0.3	1.8±0.2	877±69
α_1_β_2S2_-myc	9	3.4±0.2	1.7±0.2	730±43

N represents the cell number employed in each assay. The values shown are mean ± standard error.

Previously it was found that upon repeated GABA-stimulations, GABA_A_ receptors containing β_2L_ displayed a greater current run-down compared to those containing β_2S_ [11]. Since Thr^365^, a potential phosphorylation site for calmodulin dependent protein kinase II, is present in β_2L_ but not β_2S_, its possible involvement in current run-down was examined by a Thr365Ala mutation to yield the mutant construct β_2L-T365A_. When repeated receptor activation was induced by exposure of cells transiently expressing α_1_β_2L-T365A_γ_2L_, α_1_β_2L_γ_2L_ or α_1_β_2S_γ_2L_ to 300 µM GABA in the absence of ATP infusion (Figure 1C), Figure 1D show that the 64.3% run-down reduction relative to initial current displayed by the β_2L-T365A_-containing receptors was smaller than the 77.3% reduction displayed by the wildtype β_2L_-containing receptors, and practically the same as the 64.0% reduction displayed by the β_2S_-containing receptors. These findings suggest that the greater sensitivity of α_1_β_2L_γ_2L_ relative to α_1_β_2S_γ_2L_ toward current run-down is caused by the depletion of intracellular ATP affecting the phosphorylation status of Thr^365^. In keeping with this, addition of 4 mM ATP to the intracellular infusate reduced the run-down amplitudes of all three kinds of receptors, and effectively narrowed the differences between them (Figure 1E and Figure S6).

### Genetic correlations with isoform expressions in psychotic disorders

**Figure 4:**
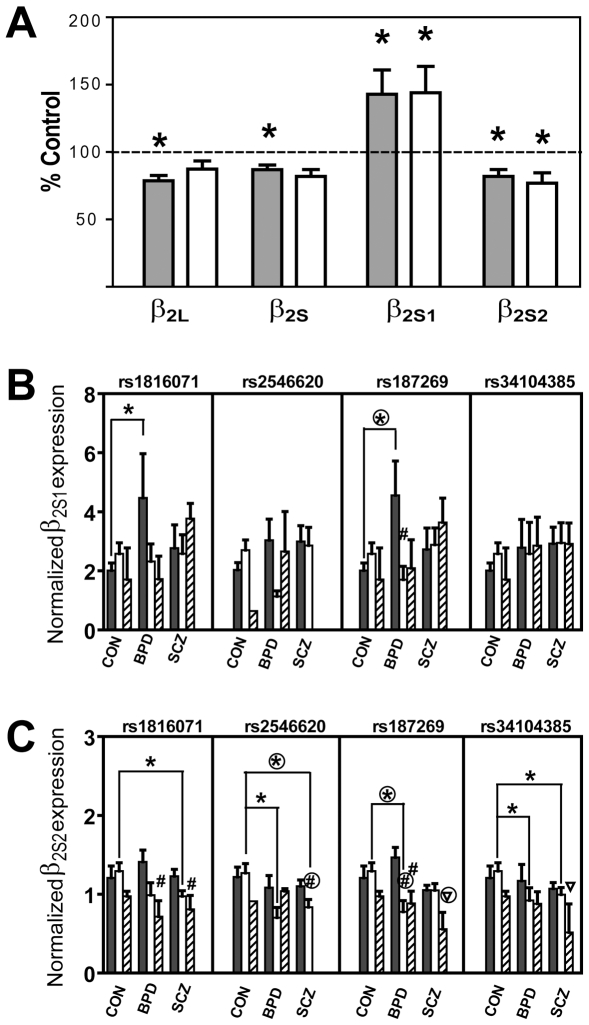
Correlations of isoform expressions with disease and genotype. (A) Expressions of β_2L_, β_2S_, β_2S1_ and β_2S2_ in SCZ and BPD relative to CON. The mean expressions of each of the four isoforms in SCZ are represented by shaded columns, and the mean expressions in BPD by clear columns. The CON level is represented by the horizontal dashed line. Significant deviations (*P*<0.05) of mean expressions in SCZ or BPD from CON, examined using UNPHASED, is marked by * above columns. Relative expressions of β_2S1_ (part B) and β_2S2_ (part C) are shown for the homozygous-major MM (grey), heterozygous Mm (white), and homozygous-minor mm (hatched) genotypes of the tagging SNPs rs1816071, rs2546620, rs187269, and rs34104385 in the male CON, SCZ and BPD cohorts. Standard errors are indicated by bars. Expression levels in BPD or SCZ that deviated significantly from CON based on ANOVA with LSD *post hoc* test are marked by * above the indicated comparison. Any expression level of Mm or mm that deviated significantly from MM within the diagnostic group is marked by #. Any expression level of mm that deviated significantly from both MM and Mm is marked by∇. After additional testing by the Bonferroni *post hoc* test, those cases remaining significant (*P*<0.05) were marked as circled *, # or ∇.

Expressions of the four isoforms β_2L_, β_2S_, β_2S1_ and β_2S2_ in DLPFC of postmortem CON, SCZ and BPD brains were determined using quantitative real-time PCR, and normalized by the geometric mean of the three reference genes glyceraldehyde-3-phosphate dehydrogenase (*GAPDH*), ubiquitin C (*UBC*) and hydroxymethyl-bilane synthase (*HMBS*) as described [11]. The levels of β_2S1_ were significantly increased in both SCZ (by 43.0%, *P_LRS_* = 0.038) and BPD (by 43.7%, *P_LRS_* = 0.031) (Figure 4A), whereas the levels of β_2S2_ were significantly decreased in both SCZ (by 17.9%, *P_LRS_* = 0.036) and BPD (by 22.6%, *P_LRS_* = 0.034). All these cases also passed the global significance test with 1,000 permutations. These observations on β_2S1_ and β_2S2_ were compared to earlier results on β_2S_ and β_2L_ [11] in Figure 4A, which showed that the mean expression levels of all four isoforms in SCZ deviated significantly from the CON levels. The mean expression levels in BPD deviated from the CON levels for β_2S1_ and β_2S2_, but not for β_2S_ and β_2L_. When the sample characteristics of gender, age, brain pH, postmortem interval (PMI), and sample refrigeration interval (RFI) were included individually as a covariate in UNPHASED analysis, the Wald test indicated no significant covariate effect on β_2S1_ or β_2S2_ expression.

Previously the SNPs and haplotypes in the Intron 8-Intron 9 region (Figure S7) have been associated with SCZ [8], [11]. The results in Table S1 extended their associations to BPD. Correlation analysis between the four isoform expressions and different alleles, haplotypes and genotypes of the six tagging SNPs in the combined CON-BPD-SCZ cohorts revealed significant correlations for β_2L_ or β_2S_ at the allelic, genotypic as well as haplotypic levels (Table 2). When the males were analyzed in separation from the females, comparable significant correlations were also observed with β_2S2_ expression. The various effects relating to isoform expressions involved particularly the tagging-SNPs rs1816071, rs252944, rs187269, and rs34104385, all of which were significantly associated with both BPD (Table S1) and SCZ [8], [11].

**Table 2:** The relationships of alleles, genotypes and haplotypes with isoform expressions in the combined CON, BPD and SCZ cohorts.

	rs1816071	rs2546620	rs13178374	rs252944	rs187269	rs34104385	Male+Female				Male			
							β_2L_	β_2S_	β_2S1_	β_2S2_	β_2L_	β_2S_	β_2S1_	β_2S2_
Alleles	**X**						**0.010**	**0.010**	0.833	0.080	**0.005**	**0.005**	0.929	**0.002**
					**X**		**0.046**	0.055	0.860	0.084	**0.027**	**0.023**	0.720	**0.010**
Genotypes	**X**						**0.023**	**0.024**	0.883	0.147	**0.016**	**0.021**	0.960	**0.005**
				**X**			**0.030**	**0.019**	0.680	0.056	0.192	0.252	0.542	0.192
					**X**		**0.041**	0.045	0.827	0.151	0.053	0.065	0.918	**0.022**
Haplotypes	**X**	**X**					**0.032**	**0.035**	0.966	0.127	**0.018**	**0.018**	0.733	**0.005**
	**X**		**X**				**0.033**	**0.036**	0.960	0.161	**0.018**	0.019	0.672	**0.006**
	**X**			**X**			**0.031**	**0.030**	0.500	0.184	**0.013**	**0.017**	0.706	**0.004**
	**X**				**X**		0.080	0.081	0.936	0.320	0.039	0.043	0.884	**0.013**
	**X**					**X**	**0.006**	0.036	0.305	0.272	**0.002**	**0.022**	0.256	**0.012**
		**X**			**X**		0.130	0.160	0.861	0.093	0.088	0.076	0.848	**0.032**
			**X**		**X**		0.097	0.092	0.981	0.221	**0.029**	0.092	0.750	**0.019**
			**X**			**X**	0.582	0.275	0.792	0.459	0.457	0.327	0.599	**0.025**
					**X**	**X**	**0.019**	0.123	0.339	0.244	**0.008**	0.073	0.337	0.062

The X in each column indicates the SNP-composition of the one-SNP alleles, genotypes, or two-SNP haplotypes in the combined CON, BPD and SCZ cohorts in that row. *P_LRS_*-values shown in the table were obtained from the likelihood ratio test for correlations between allele or haplotype on the one hand, and expression of β_2L_, β_2S_, β_2S1_ or β_2S2_ on the other. Global tests with 1000 permutations were applied to all the significant cases (*P*_LRS_<0.05) to yield *P*_Glob_, and only those *P*_LRS_ values that yielded both *P*_LRS_<0.05 and *P*_Glob_<0.05 are shown in bold font in the Table.

The relationships between isoform expressions and individual genotypes of the six tagging SNPs were further analyzed in the male samples using ANOVA both between and within diagnostic groups (Figure 4B–4C and Table S2). The interdiagnostic group comparisons with LSD tests showed that the MM genotypes of rs1816071 (*P* = 0.017) and rs187269 (*P* = 0.009) significantly increased β_2S1_ in BPD relative to CON (Figure 4B, indicated by *). On the other hand, the Mm genotypes of rs1816071 (*P* = 0.028), rs2546620 (*P* = 0.010) and rs34104385 (*P* = 0.047) significantly reduced β_2S2_ in SCZ, and the Mm genotypes of rs2546620 (*P* = 0.037), rs187269 (*P* = 0.004) and rs34104385 (*P* = 0.047) significantly reduced β_2S2_ in BPD (Figure 4C, indicated by *). When additionally tested by the Bonferroni *post hoc* test, the significant correlations between MM of rs187269 and increased β_2S1_ in BPD, between Mm of rs2546620 and decreased β_2S2_ in SCZ, and between Mm of rs187269 and decreased β_2S2_ in BPD, all remained significant at *P*<0.05 (as indicated by circled * in Figure 4B–4C).

The intradiagnostic group comparisons showed that in the BPD cohort, the Mm genotype of rs187269 significantly decreased β_2S1_ expression relative to the MM genotype (*P* = 0.022) (Figure 4B, indicated by #); the mm genotype of rs1816071 significantly decreased β_2S2_ expression relative to MM (*P* = 0.026) (Figure 4C, indicated by #); and the Mm (*P* = 0.004) and mm (*P* = 0.033) genotypes of rs187269 both significantly decreased β_2S2_ expression relative to MM. In the SCZ cohort, the mm genotype of rs1816071 significantly decreased β_2S2_ expression relative to MM (*P* = 0.029); the Mm genotype of rs2546620 significantly decreased β_2S2_ expression relative to MM (*P* = 0.044); and the mm genotypes of rs187269 and rs34104385 both significantly decreased β_2S2_ expression relative to the MM as well as Mm genotypes (all *Ps*<0.05) (Figure 4C, indicated by∇). When additionally tested by the Bonferroni *post hoc* test, some of these correlations remained significant at *P*<0.05 (as indicated by circled # or ∇ in Figure 4C). Various *P* values are given in Table S2.

## Discussion

### Novel isoforms in a splicing hot-spot

The β_2_ subunit polypeptide of the GABA_A_ receptors, encoded by up to eleven exons, is known to exist in different isoforms: β_2L_ which contains Exon 10, and β_2S_ which is devoid of Exon 10 [5]. Since the β_2L_ and β_2S_ expressions are associated with SCZ, and also produce different effects on GABA_A_ receptor run-downs, they could provide a fundamental link between *GABRB2* genotypes and physiological perturbations in schizophrenia [11], [15]. In the minigene system, while minigene Δ2 gave rise to an Exon 10-containing Band II and an Exon 10a-containing Band I, a shortening of Δ2 to Δ3 that included the removal of the gene expression-correlated and schizophrenia-associated SNP rs187269 [8] abolished both Band II and Band I. Likewise, other minigene deletions gave rise to Exon 10b (expressed from Δ4) and Exon 10c (expressed from Δ5). These findings pointed to the facile occurrence of different splicing modes in the Exon 9-Exon 11 segment in response to intron sequence variations. They prompted a search among human brain cDNAs for novel splicing products and led to the discovery of the two novel β_2_ isoforms β_2S1_ and β_2S2_ in the brain. In the β_2S1_ isoform, Exon 9 is extended through Intron 9 retention, and both Exons 3, 10 and 11 were omitted (Figure 3A). In the β_2S2_ isoform, Exon 10 is omitted, and Exon 11 is shortened by a 64-bp deletion that brought about a frameshift-generated TAG. The absence of Exon 11 from β_2S1_ and β_2S2_ implies the loss of the C-terminal transmembrane domain TM4 from these β_2_ isoforms. Functionally, the different β_2_ isoforms displayed non-identical EC_50_ values. Since the EC_50_ values of β_2S_ (containing Exon 11) and β_2S1_ (devoid of Exon 11) were not greatly different, Exon 11 and its constituent TM4 domain might not be an important determinant of EC_50_. Similarly, since the EC_50_ of β_2L_ (containing Exon 10) was also not greatly different from those of β_2S_, β_2S1_ and β_2S2_ (all devoid of Exon 10), Exon 10 also might not be an important determinant of EC_50_. That the β_2S2_ isoform was not detected earlier was likely due to its similar size as β_2S_, differing only by 64-bp, such that β_2S2_ was only found through screening of individual clones bearing RT-PCR products. The β_2S1_ isoform on the other hand has hitherto escaped detection probably because it became detectible only using PCR primers suggested by the minigene results.

The existence of as many as four isoforms, viz. β_2S_, β_2S2_, β_2S1_ and β_2L_ arising from alternative splicings of the Exon 10 region suggests that this region represents an ‘alternative-splicing hotspot’. The brain-region distributions of β_2S_, β_2L_ and β_2S2_ were largely similar whereas that of β_2S1_ was more dissimilar. Exons 10a, 10b and 10c, which were expressed only by minigenes (viz. Δ2, Δ4 and Δ5 respectively) that carried a deletion in Intron 9, were not detected in the brain.

### Relationships between novel isoforms and psychotic disorders

Based on the six tagging SNPs in the Intron 8-Intron 9 segment, the results in Table 2 showed that the expression of the novel β_2S2_ isoform participated, at least among the males, along with the expressions of β_2S_ and β_2L_ in a number of allelic, genotypic and haplotypic correlations. As shown in Figure 4A, the expressions of all four isoforms in SCZ deviated significantly from CON, and this was also the case with β_2S1_ and β_2S2_ in BPD. Thus a derangement in β_2_ isoform expressions represents an important characteristic common to these two major psychotic disorders.

The genotypic effects recorded for the MM, Mm and mm genotypes of rs187269 in Figure 4B showed generally higher levels in the male BPD and SCZ cohorts compared to CON in agreement with the overall increases in β_2S1_ in BPD and SCZ (Figure 4A), with the positive correlations between β_2S1_ in BPD and genotype reaching the *P*<0.05 significance level in the case of rs187269 MM (Figure 4B). On the other hand, the genotypic effects showed generally lower levels in the BPD and SCZ cohorts compared to CON in agreement with the overall decreases in β_2S2_ in BPD and SCZ (Figure 4A), with the negative correlations between β_2S2_ in SCZ and genotype reaching the *P*<0.05 significant level in the case of the rs2546620 Mm and negative correlations between β_2S2_ in BPD and genotype also reaching the *P*<0.05 significant level in the case of the rs187269 Mm (Figure 4C). Thus the observed increases in β_2S1_ expression in BPD in Figure 4A were led by increased expression by the MM genotype more so than any increased expression by the Mm or mm genotype, whereas the observed decreases in β_2S2_ expression in BPD and SCZ were led by decreased expression by the Mm genotype more so than any decreased expression by the MM or mm genotype. Correspondingly, such genotype-dependent alterations in β_2S2_ expression were supported by the statistically significant inter-genotype differences (marked by # and ∇ in Figure 4C) observed in the BPD and SCZ cohorts, in contrast to the absence of such differences in the CON cohort.

In conclusion, β_2S1_ and β_2S2_, the two novel isoforms of GABA_A_ receptor β_2_-subunit discovered in the present study, shared two fundamental attributes with the previously known β_2S_ and β_2L_ isoforms: the expressions of all four were significantly correlated with not only SCZ or BPD or both, but also with the genotypes of SNPs in the Intron 8-Intron 9 region of the *GABRB2* gene. The present study therefore has rendered even more unambiguous the importance of the SNPs in this region of *GABRB2* with respect to isoform expressions and the etiologies of the two major psychotic disorders.

## Materials and Methods

### DNA and RNA samples

RNA was extracted from tissue culture cells using guanidine method [16]. Brain RNA samples from mouse, rhesus monkey, and different human brain regions were purchased from BioChain Institute. Postmortem brain DNA and RNA of 31 SCZ, 30 BPD and 31 CON were donated by the Stanley Collection (Table S3) [17]. After treatment with DNase I (Invitrogen), RNA was reverse transcribed to cDNA. Reaction was carried out with 2 µg of total RNA, random hexamers and TaqMan Reverse Transcription Kit (Applied Biosystems) for 10 min at 25°C, 30 min at 48°C, 5 min at 95°C.

### Oligonucleotide primers and probes

The primers for PCR, DNA sequencing and cloning, and probes of *GABRB2* isoforms and *GAPDH* for real-time PCR were designed using Primer Express 3.0 (Applied Biosystems Inc., Foster City, CA), as described in Table S4, and obtained from Proligo Singapore Pte Ltd. TaqMan Expression Assays (Applied Biosystems) were employed for *UBC* and *HMBS*.

### Minigene construction

A fragment that spanned from the start of Exon 9 to 1207 bp downstream of Exon 10 of *GABRB2* was amplified from human genomic DNA with the addition of a GCTCTAGA sequence (where the underlined sequence represents an XbaI site) to its 3′ end. This amplified fragment was cloned into the pMD18-T vector to yield pMD18T-I. Another fragment spanning from 1127 bp upstream of Exon 11 to the end of Exon 11 of *GABRB2* was likewise amplified from human genomic DNA with the additions of a GCTCTAGA (where the underlined sequence represents an XbaI site) to both its 5′ and 3′ terminals. This amplified fragment was cloned into the pMD18T-I via the XbaI site to yield pMD18T-I/II, such that the cloned insert contained sequentially Exon 9, Intron 9, Exon 10, a shortened Intron 10, and Exon 11. The insert in pMD18T-I/II was in turn digested out with ApaI (located in Exon 9) and Xho I (in Exon 11), and inserted into the corresponding sites in Exon 9 and Exon 11 of the pcDNA3.1-β_2S_ construct, which carries the coding region of β_2_ cDNA without any intron. The resultant human GABA_A_ receptor β_2_ minigene was designated as WT: it contained sequentially part of Exon 2, Exons 3–9 (coding region only and devoid of Introns 2–8), Intron 9, Exon 10, an Intron 10 shortened by a 29.6 Kb deletion (bp 160,655,142–160,684,744 inclusive on Chromosome 5) and Exon 11 (Figure 2A). A series of deletions of WT were performed using the QuikChange site-directed mutagenesis kit (Stratagene, La Jolla, CA) to yield Δ1 (with a deletion of bases 3513 to 3630 inclusive of Intron 9), Δ2 (with a deletion of bases 1451 to 3630 inclusive of Intron 9), Δ3 (with a deletion of bases 1000 to 3630 inclusive of Intron 9), Δ4 (with a deletion of bases 518 to 3630 inclusive of Intron 9), Δ5 (with a deletion of bases 72 to 3630 inclusive of Intron 9), Δ6 (with a deletion of bases 72 to 2818 inclusive of Intron 9), Δ7 (with a deletion of bases 72 to 1880 inclusive of Intron 9), and Δ8 (with a deletion of bases 72 to 981 inclusive of Intron 9). WT and its various deletions were transfected into HEK293 cells, and expression of *GABRB2* mRNA in the cells was monitored using RT-PCR using the primers located in Exon 9 and Exon 11.

### Expression constructs

The coding regions of the human GABA_A_ receptor α_1_, β_2S_, β_2L_ and γ_2L_ subunit cDNAs were amplified from human brain cDNA libraries (Resgen, Invitrogen), and individually cloned into the mammalian expression vector pcDNA3.1 (Stategene, USA) as described previously [11]. The site-directed mutant pcDNA3.1-β_2L-T365A_ was generated with the PCR-based Mutagenesis Kit from Stratagene. To facilitate protein detection, the Myc tag was fused into the C-terminal of the β_2L_, β_2S_, β_2S1_ and β_2S2_ subunits by cloning into pCI-neo-Myc expression vector (Promega). Briefly, the flanking restriction sites EcoR I and Xha I were added on respectively to the 5′ terminal and 3′ terminal of the β_2L_, β_2S_, β_2S1_ and β_2S2_ cDNAs by PCR amplification. After digestion with EcoR I and Xba I, the cDNAs were inserted into the corresponding sites in pCI-neo-Myc vector. The fidelity of the final expression constructs were verified by DNA sequencing.

### Cell cultures and transfections

HEK293 cells were maintained in Dulbecco's modified Eagle's medium supplemented by 10% fetal bovine serum (Invitrogen), and transfected using Lipofectamine & Plus reagents (Invitrogen) following the manufacturer's instructions. Transfection was performed either in 35 mm dishes with 3 µg/dish plasmid DNA, or in 12-well plates with 1.2 µg/well plasmid DNA. Cells were employed for RNA extraction, immunostaining and electrophysiology 48 h after transfection.

### Properties of recombinant GABA_A_ receptors

For immunostaining, cells plated on poly-L-lysine coated coverslips were fixed in paraformaldehyde. After blocking with bovine serum albumin and anti-Myc antibody (9E10 from mouse, diluted 1∶500), fluorescein-conjugated goat anti-mouse IgG (from goat and ditulted 1∶100; Molecular Probe) was applied, and immunostaining was examined using a Nikon TE2000E fluorescence microscope.

The recording of GABA dose-current responses in HEK293 cells containing recombinant receptors by means of whole-cell patch-clamping, and the induction of receptor run-down by repeated GABA-stimulations, was performed as described [11], [18]. Variation of current with GABA concentration was fitted to the Hill equation to estimate EC_50_ and Hill coefficient (n_H_).

### Quantitative PCR and DNA sequencing

Quantitative real-time PCR was performed with TaqMan Probe as described [11] (Table S4). The geometric mean of three reference genes *GAPDH*, *HMBS* and *UBC* was employed for normalization of *GABRB2* expression [19].

Genotypes of various SNP and of a 12-bp sequence at rs34104385 were determined by sequencing of a PCR-amplified 4,469 bp segment spanning from 2,185 bp upstream to 2,039 bp downstream of Exon 9 of *GABRB2* (Figure S7), as described [11].

### Statistical analysis

Sequence alignments of *GABRB2* gene among different species were downloaded from UCSC Genome Browser website. Analyses of disease association of SNPs and two-SNP haplotypes, and analyses of correlations between isoform expression and disease status, allele, genotype or haplotype, were carried out with the likelihood ratio statistic (LRS) test to generate the *P*_LRS_ value using UNPHASED program version 3.0.7 [11], [20]. Covariate analyses of demographic or clinical characteristics were performed using the Wald-test in UNPHASED v3.07; each case showing *P*_LRS_<0.05 was further evaluated by the LRS test with 1,000 permutations to generate a global *P_Glob_* value. Correlations between isoform expression and genotype were analyzed using ANOVA with LSD and Bonferroni *post hoc* tests in SPSS v11.5.

## Supporting Information

Figure S1 Sequence alignments of (A) Exon 9 and (B) Exon 11 from different vertebrate species were downloaded from UCSC genome browser (http://genome.ucsc.edu/cgi-bin/hgGateway).
(0.51 MB JPG)

Figure S2 The full-length β_2L_ coding sequences from mouse (A) and monkey (B) brain cDNA libraries. RT-PCRs were performed with the three forward primers and three reverse primers given in Table S4, and the products were cloned into T-vector. The positive clones were validated by DNA sequencing.
(0.70 MB JPG)

Figure S3 Intron sizes of *GABRB2* in vertebrate species.
(0.20 MB JPG)

Figure S4 The cDNA sequences of Bands I, II, III, IV and V shown in Figure 2 determined by cloning the cDNA in pMD-18T vector and sequencing. Band I cDNA was obtained from Δ2, Band II from human brain, Δ2 and Δ5, Band III from Δ5, Band IV from Δ4, and Band V from all of human brain, WT and Δ1-Δ8. The dashed arrows represent the PCR primer binding regions used in RT-PCR. The boxed sequences indicate the exon (Exon 10, Exon 10a, Exon 10b or Exon 10c) found in each instance between Exon 9 and Exon 11. There was no such exon in the case of Band V. The slash within the novel exons in I, III, IV indicates in each instance the gapped position in the minigene construct.
(0.46 MB JPG)

Figure S5 GABA concentration-response currents (A) and curves (B) from transfected HEK293 cells expressing different isoforms of β_2_ together with α_1_ subunits. Recordings of representative current response of different doses of GABA are illustrated in part A. In part B, from left to right at 50% I_max_ (marked by horizontal dotted line): α_1_β_2S_-Myc, α_1_β_2S1_-Myc, α_1_β_2L_-Myc and α_1_β_2S2_-Myc, each representing the average estimate obtained with 5-9 cells through data fitted to the Hill equation.
(0.30 MB DOC)

Figure S6 GABA-potentiated current rundowns of GABA_A_ receptors. Repeated GABA_A_ receptors activation was reduced by exposure of cells transiently expressing α_1_, γ_2_ plus one of the β_2L-T365A_, β_2L_, or β_2S_ to 300 µM of GABA in the presence of 4 mM of ATP infusion
(0.11 MB JPG)

Figure S7 The positions of SNPs in the Intron 8-Intron 9 of *GABRB2* examined in the present study. SNP rs34104385 is a polymorphic site consisting of a 12-bp insertion-deletion variation (indel) followed by a A/C SNP, where the major A-allele is linked to the deletion form, and the minor C-allele is linked to the insertion form.
(0.18 MB JPG)

Table S1 *P*_LRS_-values relating to association of single SNPs and two-SNP haplotypes of *GABRB2* with male BPD and SCZ cohort. The SCZ vs CON results, with the exception of those relating to rs34104385, were based on Ref. 11. The *P*_LRS_-values shown were obtained with UNPHASED 3.07. Only those *P*_LRS_<0.05 further confirmed to be significant after 1,000 permutations are shown in bold font.
(0.01 MB DOC)

Table S2 Genotypic correlations with expressions of β_2S1_ and β_2S2_ in the male SCZ, BPD and CON cohorts. Genotypic effects of the tagging SNPs rs1816071, rs2546620, rs187269 and rs34104385 on β_2S1_ and β_2S2_ expressions are shown for the male SCZ, BPD and CON cohorts. N represents the number of samples of each of the three genotypes MM, Mm and mm. Overall genotypic effects on β_2S1_ and β_2S2_ expressions were assessed using ANOVA. Pair-wise genotype comparisons (MM vs. Mm, Mm vs. mm and MM vs. mm) were evaluated with LSD post hoc test in ANOVA, and the results were shown as *P*_L_; the instances with *P*_L_<0.05 were further evaluated with Bonferroni post hoc test in ANOVA, and the results were shown as *P*_B_. In all instances, *P*<0.05 values are shown in bold font. The three Mean Ratios for each group refer to the expression level of, from top to bottom, the ratio of MM over Mm, Mm over mm, and MM over mm.
(0.09 MB DOC)

Table S3 Demographic and clinical information on subjects used in this study. Mean ± standard deviation; M, male; F, female; PMI, postmortem interval; BW, brain weight; RFI, duration of refrigerator storage; AOO, age of onset; TIH, time in hospital; DOI, duration of illness; LFPZ, lifetime of antipsychotic exposure in terms of fluphenazine equivalent in grams. ^a^35 CON, 35 SCZ and 35 BPD were provided by Stanley Research Foundation and 13 from three groups giving no RT-PCR products were excluded from this study. ^b^RNA sample obtained from BioChain Institute included one from rhesus monkey cortex, and 17 different human brain regions, with age of individual in parenthese: cerebral cortex (23), cerebellum (29), cerebral meninges (21), corpus callosum (27), diencephalon (29), postcentral gyrus (41), precentral gyrus (26), hippocampus (82), frontal lobe (41), occipital lobe (27), parietal lobe (26), temporal lobe (26), medulla oblongata (60), olfactory (87), pons (36), pituitary (53) and thalamus (71).
(0.01 MB DOC)

Table S4 Primers and probes used in this study. Restriction sites are underlined.
(0.02 MB DOC)
