# Supplementary figures and images for: Alternative-Splicing in the Exon-10 Region of GABAA Receptor β2 Subunit Gene: Relationships between Novel Isoforms and Psychotic Disorders
Source: PLoS One. 2009 Sep 18;4(9):e6977. doi: 10.1371/journal.pone.0006977 (PMC2741204; doi:10.1371/journal.pone.0006977)

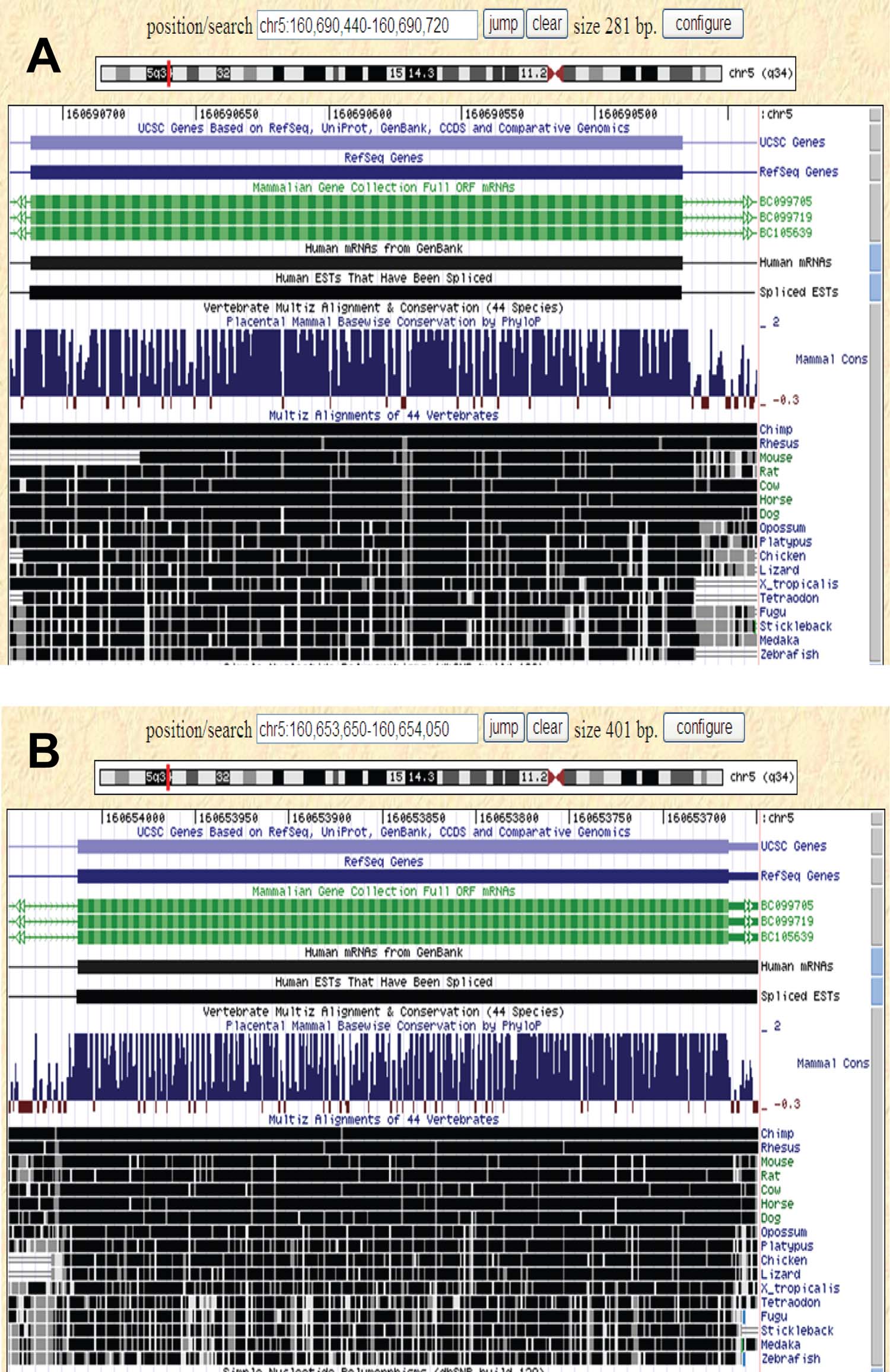

Supplement: Figure S1 — Sequence alignments of (A) Exon 9 and (B) Exon 11 from different vertebrate species were downloaded from UCSC genome browser (http://genome.ucsc.edu/cgi-bin/hgGateway). (0.51 MB JPG) [file pone.0006977.s001.jpg]

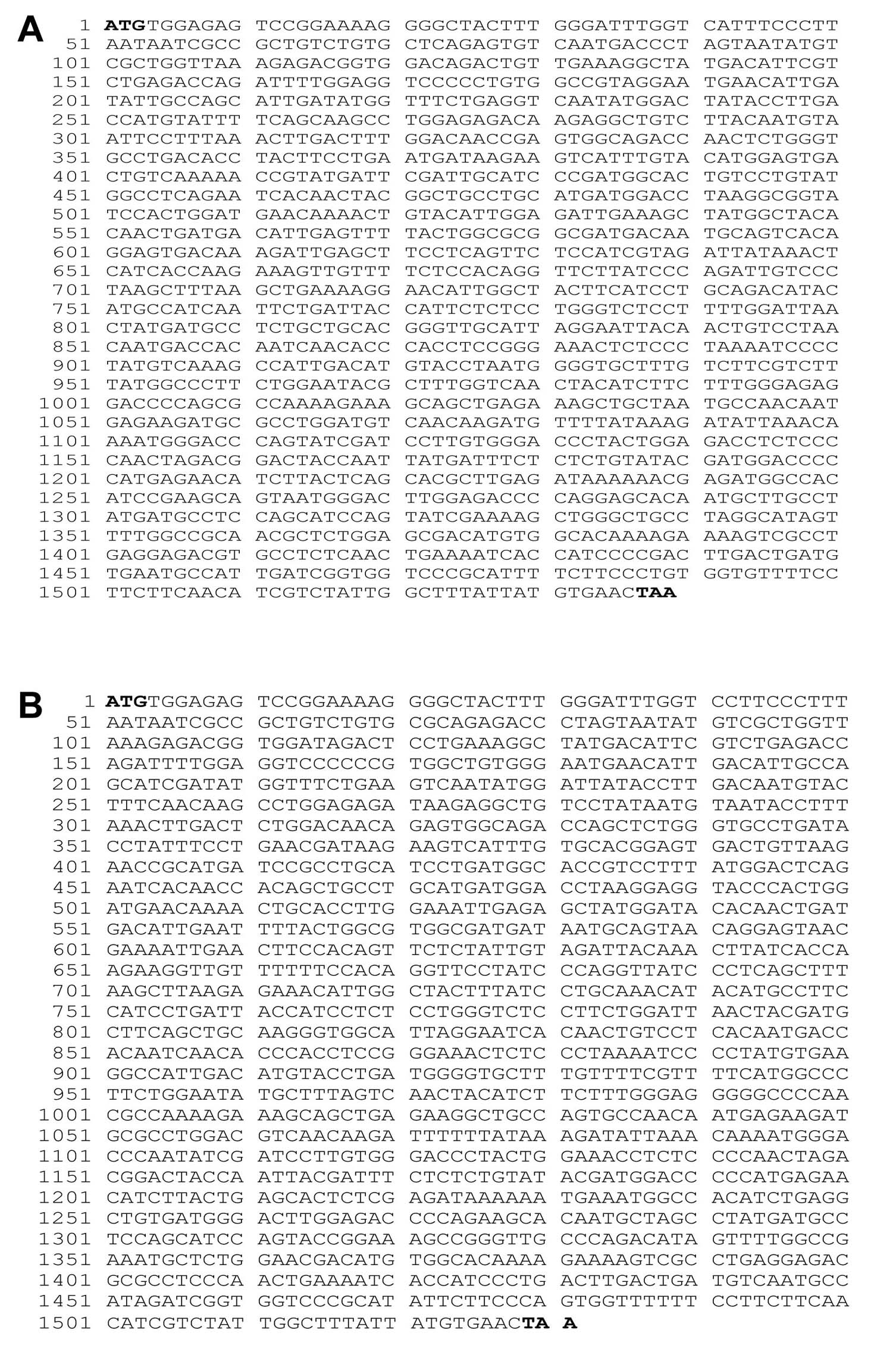

Supplement: Figure S2 — The full-length β2L coding sequences from mouse (A) and monkey (B) brain cDNA libraries. RT-PCRs were performed with the three forward primers and three reverse primers given in Table S4, and the products were cloned into T-vector. The positive clones were validated by DNA sequencing. (0.70 MB JPG) [file pone.0006977.s002.jpg]

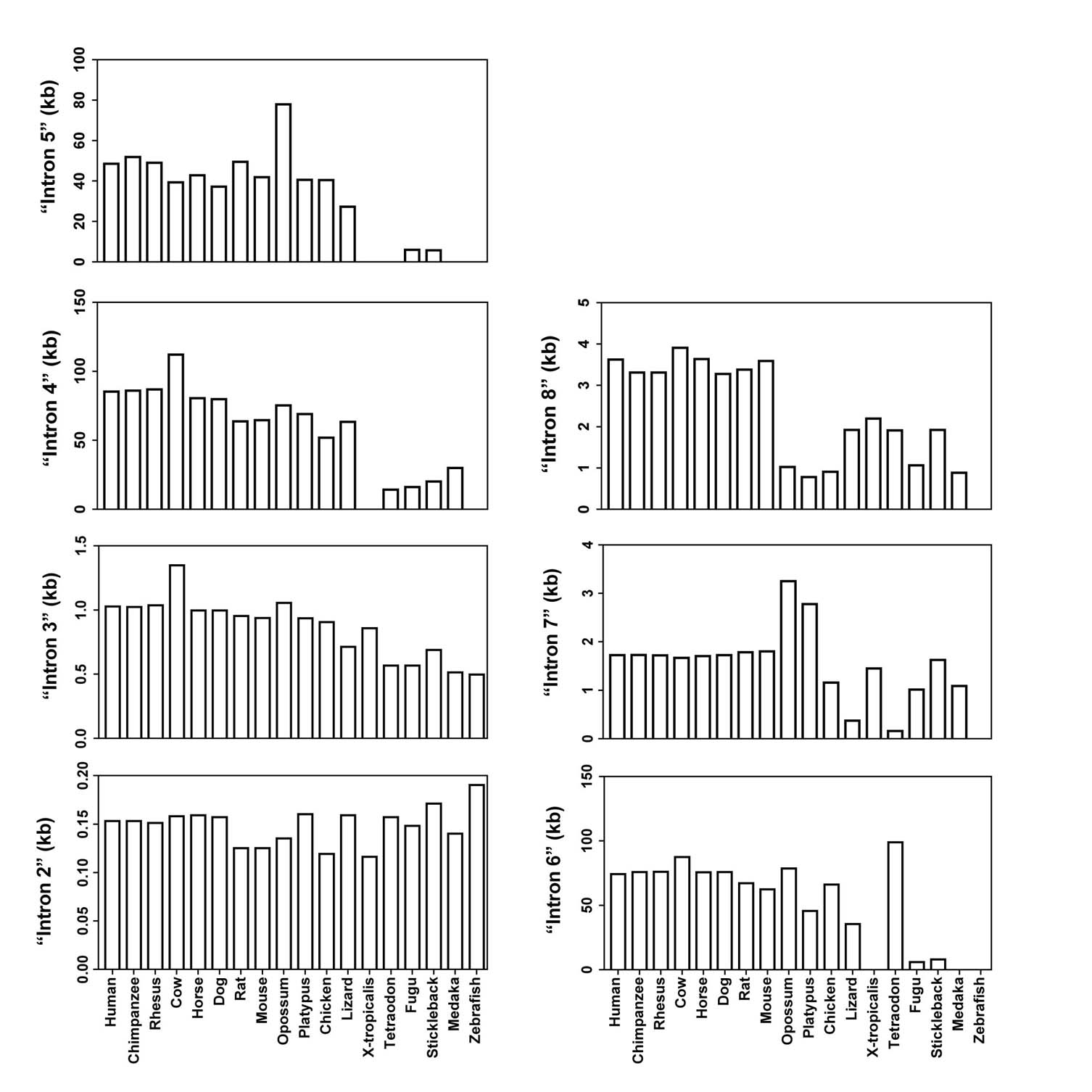

Supplement: Figure S3 — Intron sizes of GABRB2 in vertebrate species. (0.20 MB JPG) [file pone.0006977.s003.jpg]

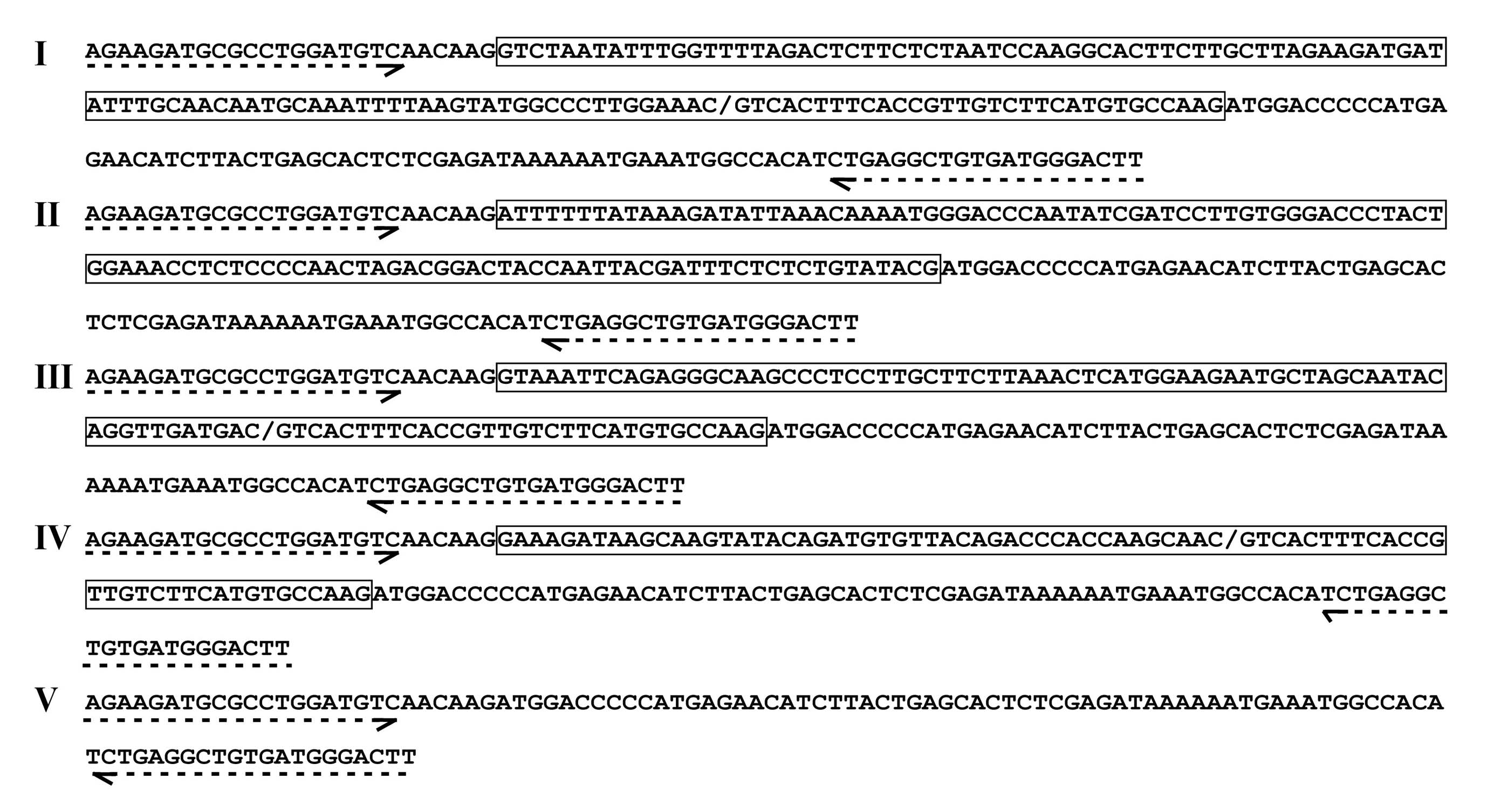

Supplement: Figure S4 — The cDNA sequences of Bands I, II, III, IV and V shown in Figure 2 determined by cloning the cDNA in pMD-18T vector and sequencing. Band I cDNA was obtained from Δ2, Band II from human brain, Δ2 and Δ5, Band III from Δ5, Band IV from Δ4, and Band V from all of human brain, WT and Δ1-Δ8. The dashed arrows represent the PCR primer binding regions used in RT-PCR. The boxed sequences indicate the exon (Exon 10, Exon 10a, Exon 10b or Exon 10c) found in each instance between Exon 9 and Exon 11. There was no such exon in the case of Band V. The slash within the novel exons in I, III, IV indicates in each instance the gapped position in the minigene construct. (0.46 MB JPG) [file pone.0006977.s004.jpg]

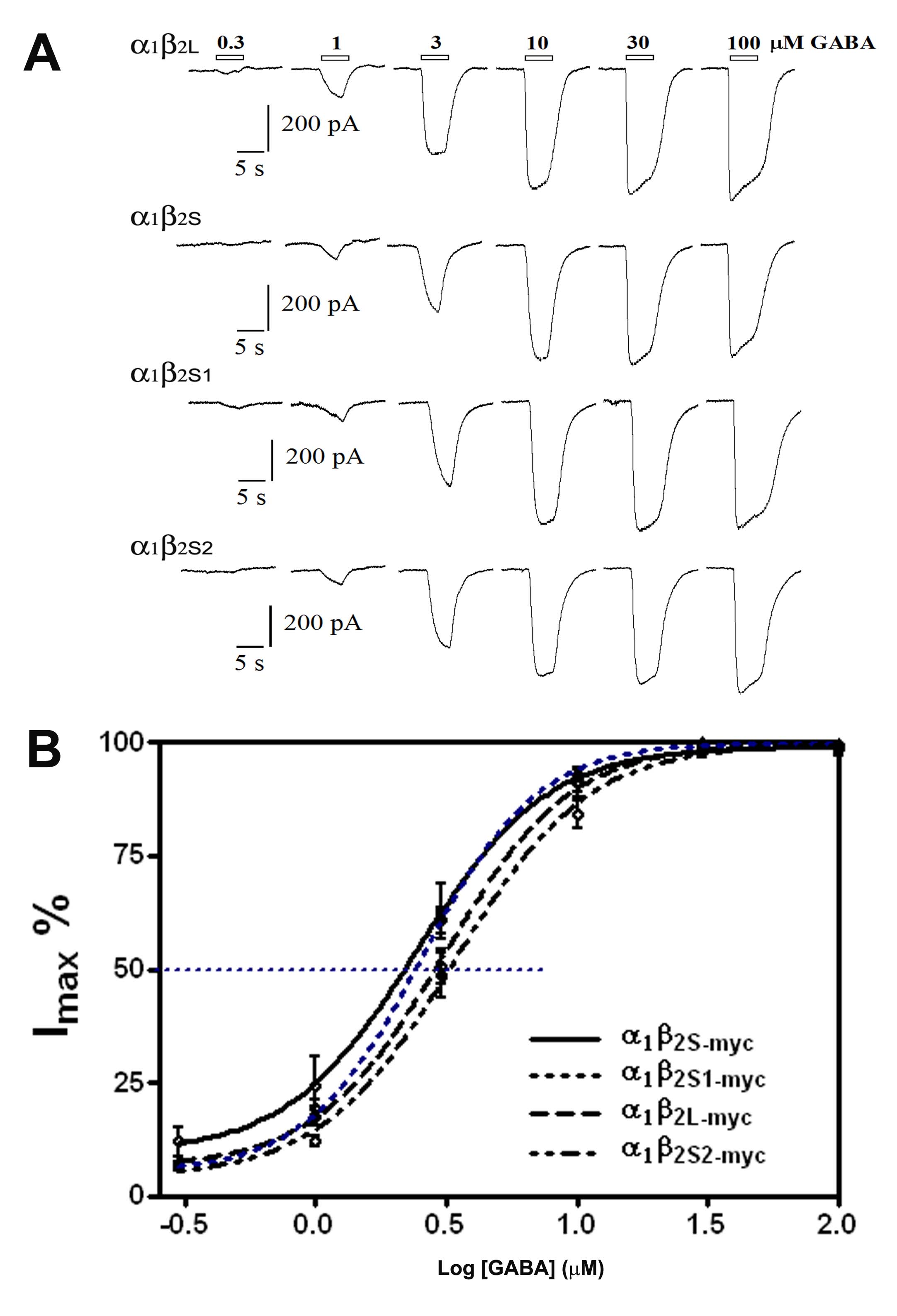

Supplement: Figure S5 — GABA concentration-response currents (A) and curves (B) from transfected HEK293 cells expressing different isoforms of β2 together with α1 subunits. Recordings of representative current response of different doses of GABA are illustrated in part A. In part B, from left to right at 50% Imax (marked by horizontal dotted line): α1β2S-Myc, α1β2S1-Myc, α1β2L-Myc and α1β2S2-Myc, each representing the average estimate obtained with 5-9 cells through data fitted to the Hill equation. (0.30 MB DOC) [file pone.0006977.s005.jpg]

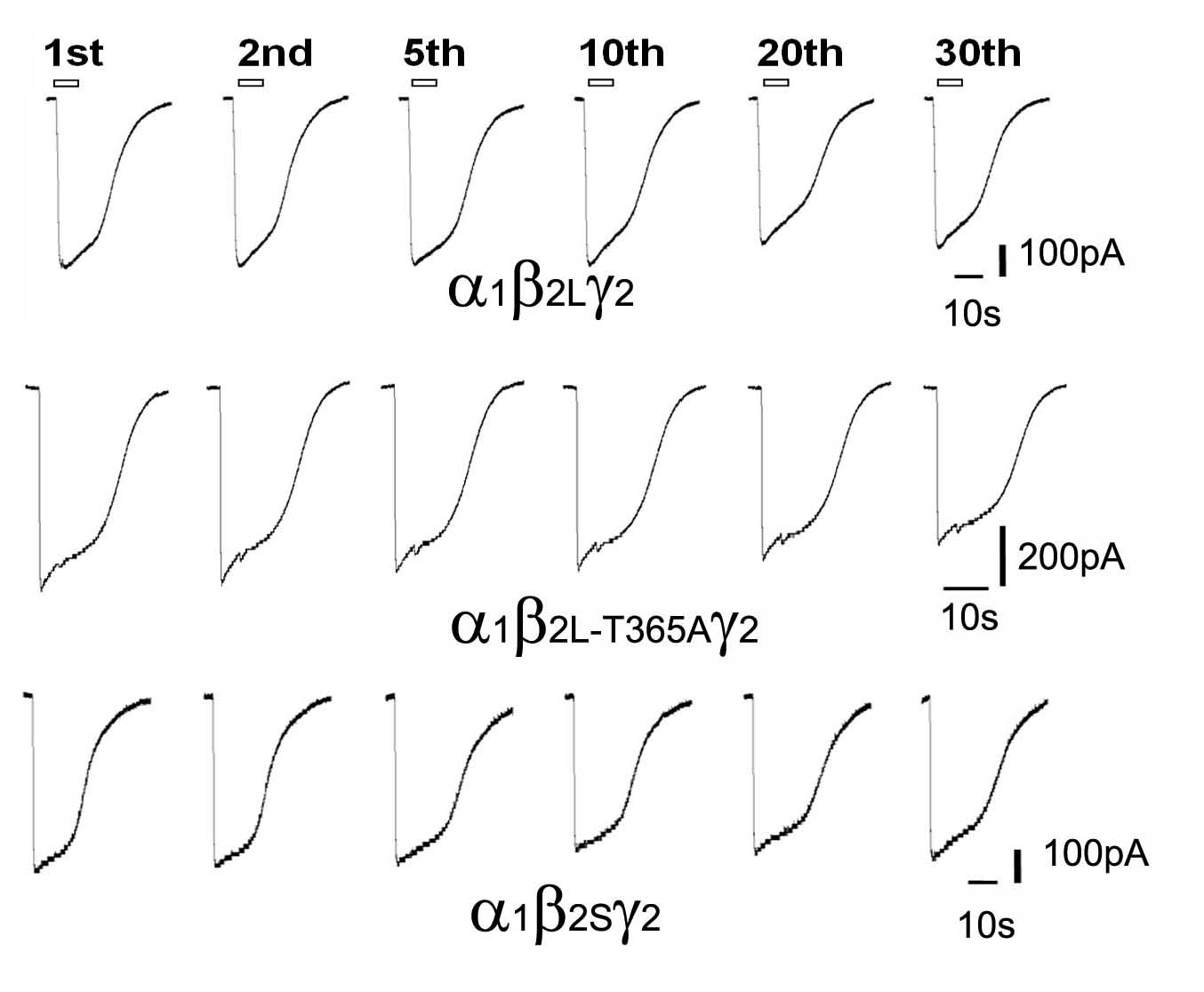

Supplement: Figure S6 — GABA-potentiated current rundowns of GABAA receptors. Repeated GABAA receptors activation was reduced by exposure of cells transiently expressing α1, γ2 plus one of the β2L-T365A, β2L, or β2S to 300 µM of GABA in the presence of 4 mM of ATP infusion (0.11 MB JPG) [file pone.0006977.s006.jpg]

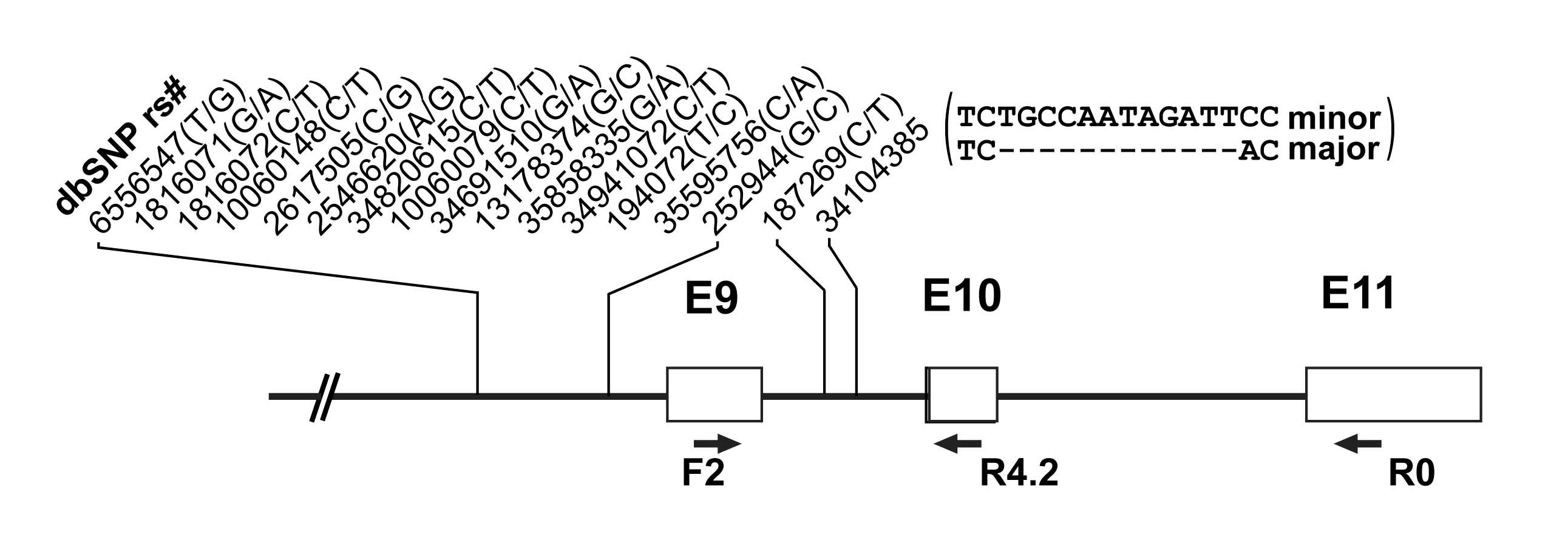

Supplement: Figure S7 — The positions of SNPs in the Intron 8-Intron 9 of GABRB2 examined in the present study. SNP rs34104385 is a polymorphic site consisting of a 12-bp insertion-deletion variation (indel) followed by a A/C SNP, where the major A-allele is linked to the deletion form, and the minor C-allele is linked to the insertion form. (0.18 MB JPG) [file pone.0006977.s007.jpg]
