# Supplementary material for: Alternative-Splicing in the Exon-10 Region of GABAA Receptor β2 Subunit Gene: Relationships between Novel Isoforms and Psychotic Disorders
Source: PLoS One. 2009 Sep 18;4(9):e6977. doi: 10.1371/journal.pone.0006977 (PMC2741204; doi:10.1371/journal.pone.0006977)
Supplement: Table S4 — Primers and probes used in this study. Restriction sites are underlined. (0.02 MB DOC) [file pone.0006977.s011.docx]

**Target/Application**

**Primer/ probe or ABI-cat.no.**

**Accession**

**No.**

**Target Region/**

**Restriction site**

**For screening novel isoforms of**

**β**

**2**

F1

TCAGTGAAACCCTCAATTCCA

Exon 2

F2

AGGCTGCCAGTGCCAACAA

Exon 9

R1

GTCATCAACCTGTATTGCTAGCATTC

Intron 9

R2

GTTGCTTGGTGGGTCTGTAA

Intron 9

R3

GTTTCCAAGGGCCATACTTAAAAT

Intron 9

R4

ATGAAGACAACGGTGAAAGTGAC

Intron 9

R4.2

AGTAGGGTCCCACAAGGATCGATA

Exon 10

R0

GTCTCCTCAGGCGACTTTTC

Exon 11

R5

TTAGTTCACATAATAAAGCCAATA

Exon 11

**For genotyping**

PCR amplication

Forward Primer

ATGGAGGAAAGGTCCATATCTAGT

NC_000005

Intron 8

Reverse Primer /218R

CAGACAATGCCTAATGTCCTCTGG

Intron 9

DNA sequencing

Reverse Primer

GCACTGAAAGTCCAAGGTCC

Intron 8

Reverse Primer

CCTAATGGGGGAGTTTGAAC

Intron 8

Reverse Primer

CTTAATAGCTGGAAAGGTGAT

Intron 8

Reverse Primer 217r

GTTTCCAAGGGCCATACTTAAAAT

Intron 9

Reverse Primer /R5

AGCTCTGCAGGTTCAGTTTG

Intron 9

**For real-time quantiative PCR (qPCR)**

β

2S1

Forward Primer

GTCTCCTTCTGGATTAATTACGATGC

Exon 8

Reverse Primer

GTCATCAACCTGTATTGCTAGCATTC

Intron 9

TaqMan Probe

FAM-TGCAAGGGTGGCATTAGGAATCACAAC-TAMRA

Exon 8, 9

β

2S2

Forward Primer

AGAAGATGCGCCTGGATGTC

Exon 9

Reverse Primer

GCAACCCAGCTTTCCGATAC

Exon 11

TaqMan Probe

FAM-TGCTGGAGGCATCATAGGCTAGTCGAGAG-TAMRA

Exon 11

*GAPDH*

Forward Primer

GAAGGTGAAGGTCGGAGTC

NM_002046

Exon 1, 3

Reverse Primer

GAAGATGGTGATGGGATTTC

TaqMan Probe

HEX-CAAGCTTCCCGTTCTCAGCC-TAMRA

*HMBS*

Hs006009297_m1

NM_000190

Exon 1, 2

*UBC*

Hs00824723_m1

NM_021009

Exon 1, 2

**For cloning full-length**

**β**

**2S**

**and**

**β**

**2L**

**from monkey and mouse cDNA library**

Forward Primer 1

AAAGGGATGTGGAGAGTCCGGAAA

Exon 2

Forward Primer 2

GGGATGTGGAGAGTCCGG

Forward Primer 3

GGGATGTGGAGAGTCCGGAAAAG

Reverse Primer 1

GTTTAGTTCACATAATAAAGCCAATAG

Exon 11

Reverse Primer 2

GTAGTTTAGTTCACATAATAAAGCCAA

Reverse Primer 3

GCTGTAGTTTAGTTCACATAATAAAGC

**For sub-cloning of**

**β**

**2**

**in pCI-neo vector with c-myc**

β

2S1

Forward Primer

G

GAATTC

ATGAACATTGACATTGCCAGC

EcoR I

Reverse Primer

GC

TCTAGA

GCATTCTTCCATGAGT

Xba I

β

2S2

Forward Primer

G

GAATTC

ATGTGGAGAGTGCGGAAAAGGG

EcoR I

Reverse Primer

GC

TCTAGA

GTCGAGAGTGCTCAGTAAG

Xba I

β

2S

and

β

2L

Forward Primer

G

GAATTC

ATGTGGAGAGTGCGGAAAAGGG

NM_000806

EcoR I

Reverse Primer

GC

TCTAGA

GTTCACATAATAAAGCCA

Xba I

**For cloning of minigene**

Forward Primer

GAATCACAACTGTCCTCAC

Exon 9

Reverse Primer

GC

TCTAGA

GATTGCCCTAGGTAATGCCCA

Xba I

Forward Primer

GC

TCTAGA

AGAGCCTATCACTGGC

Xba I

Reverse Primer

GC

TCTAGA

GTTCACATAATAAAGCCA

Xba I
