# Supplementary material for: Alternative-Splicing in the Exon-10 Region of GABAA Receptor β2 Subunit Gene: Relationships between Novel Isoforms and Psychotic Disorders
Source: PLoS One. 2009 Sep 18;4(9):e6977. doi: 10.1371/journal.pone.0006977 (PMC2741204; doi:10.1371/journal.pone.0006977)
Supplement: Supplementary file 3 [file pone.0006977.s008.docx]

SCZ vs. CON

SNP	rs1816071	rs2546620	rs13178374	rs252944	rs187269	rs34104385
Single SNP	**0.022**	0.915	**0.006**	0.485	0.217	0.894

Two-SNP haplotypes:					
rs2546620	**0.015**				
rs13178374	**0.006**	**0.022**			
rs252944	0.068	0.837	**0.016**		
rs187269	**0.002**	0.255	**0.006**	0.466	
rs34104385	**0.001**	0.643	**0.022**	0.194	0.061

BPD vs. CON

SNP	rs1816071	rs2546620	rs13178374	rs252944	rs187269	rs34104385
Single SNP	0.106	0.933	0.043	0.097	0.303	0.510

Two-SNP haplotypes:					
rs2546620	0.117				
rs13178374	0.058	0.125			
rs252944	0.179	0.335	**0.026**		
rs187269	0.058	0.401	0.058	0.252	
rs34104385	**0.002**	0.039	0.116	0.179	**0.018**
