# Supplementary material for: Alternative-Splicing in the Exon-10 Region of GABAA Receptor β2 Subunit Gene: Relationships between Novel Isoforms and Psychotic Disorders
Source: PLoS One. 2009 Sep 18;4(9):e6977. doi: 10.1371/journal.pone.0006977 (PMC2741204; doi:10.1371/journal.pone.0006977)
Supplement: Supplementary file 4 [file pone.0006977.s009.docx]

Table 2 Genotypic correlations with expressions (M)

		rs1816071						rs2546620						rs187269						rs34		
	Group	Genotypic Overall			Pairwise Genotype			Genotypic Overall			Pairwise Genotype			Genotypic Overall			Pairwise Genotype			Genotypic Overall		
		N	*F*	*P*	Mean Ratio	P_L	P_B	N	*F*	*P*	Mean Ratio	P_L	P_B	N	*F*	*P*	Mean Ratio	P_L	P_B	N	*F*	*P*
		5			1.07	0.856	-	13			1.05	0.868	-	7			0.95	0.870	-	10		
	SCZ	12	0.794	0.466	0.69	0.229	-	10	0.03	0.868				13	0.24	0.793	0.79	0.555	-	11	0.00	1.000
		6			0.74	0.398	-	0						3			0.75	0.511	-	2		
		4			1.93	0.115	-	11			2.66	0.291	-	5			2.67	**0.022**	0.066	7		
β_{2S1}	BPD	7	2.13	0.16	1.35	0.644	-	2	0.61	0.559	0.43	0.509	-	7	3.69	0.056	0.82	0.768	1.000	5	0.02	0.983
		4			2.60	0.078	-	2			1.14	0.831	-	3			2.18	0.093	0.278	3		
		12			0.78	0.215	-	14			0.75	0.092	-	12			0.78	0.215	-	12		
	CON	9	1.09	0.355	1.51	0.281	-	8	2.70	0.092	4.23			9	1.09	0.355	1.51	0.281	-	9	1.09	0.355
		2			1.18	0.698	-	1			3.19			2			1.18	0.698	-	2		
		5			1.26	0.120	0.361	13			1.32	**0.044**	**0.044**	7			1.00	0.970	1.000	10		
	SCZ	12	2.79	0.085	1.52	0.275	0.824	10	4.58	**0.044** #				13	4.00	**0.034**	1.89	**0.019**	0.057	11	3.00	0.072
		6			1.20	**0.029**	0.087	0						3			1.88	**0.013**	**0.039**	2		
		4			1.43	0.106	0.319	11			1.54	0.301	-	5			1.89	**0.004**	**0.012**	7		
β_{2S2}	BPD	7	3.31	0.072	1.39	0.282	0.846	2	0.64	0.544	0.68	0.481	-	7	6.65	**0.011**	0.88	0.650	1.000	5	0.64	0.550
		4			1.97	**0.026**	0.077	2			1.05	0.895	-	3			1.66	**0.033**	0.100	3		
