# Supplementary material for: Alternative-Splicing in the Exon-10 Region of GABAA Receptor β2 Subunit Gene: Relationships between Novel Isoforms and Psychotic Disorders
Source: PLoS One. 2009 Sep 18;4(9):e6977. doi: 10.1371/journal.pone.0006977 (PMC2741204; doi:10.1371/journal.pone.0006977)
Supplement: Supplementary file 5 [file pone.0006977.s010.docx]

Diagnosis	N	*Gender (F/M)*	*Age (year)*	*pH*	*PMI (hrs)*	*RFI (hrs)*	*AOO (year)*	*TIH (year)*	*DOI (year)*	*LFPZ (g)*
Schizophrenia (SCZ)	31[a]	8/23	43.3 ± 8.6	6.5 ± 0.2	31.4 ± 16.5	5.9 ± 4.4	21.4 ± 5.6	1.3 ± 2.4	21.8 ± 9.7	93.7 ± 103.5
Bipolar Disorders (BPD)	30[a]	15/15	44.6 ± 10.4	6.5 ± 0.3	37.8 ± 17.8	9.5 ± 7.4	25.2 ± 8.9	0.2 ± 0.3	19.4 ± 8.9	3.0 ± 6.9
Control (CON)	31[a]	7/24	43.5 ± 7.4	6.6 ± 0.3	29.0 ± 12.3	3.9 ± 2.7	-	-	-	-
Monkey (Macaca mulatta)	1[b]	0/1	10	-	-	-	-	-	-	-
Human Brain Region	17[b]	3/14	40.8 ± 20.9	-	-	-	-	-	-	-
